# The “Pollution Halo” Effect of FDI: Evidence from the Chinese Sichuan–Chongqing Urban Agglomeration

**DOI:** 10.3390/ijerph191911903

**Published:** 2022-09-21

**Authors:** Lei Gao, Taowu Pei, Jingran Zhang, Yu Tian

**Affiliations:** 1 School of Economics and Management, Yanshan University, Qinhuangdao 066004, China or; 2 College of Economics and Management, China Agricultural University, Beijing 100083, China; 3 School of Economics and Management, Beijing Forestry University, Beijing 100083, China; 4 Institute of Ancient Books, Jilin University, Changchun 130012, China

**Keywords:** FDI, PM_2.5_ pollution, pollution halo, STIRPAT model, Sichuan–Chongqing urban agglomeration

## Abstract

In this paper, panel data from nineteen key cities in the Sichuan–Chongqing urban agglomeration from 2003 to 2016 were used as the study sample. Using the stochastic impacts by regression on population, affluence, and technology (STIRPAT) model, the effect of foreign direct investment (FDI) on particulate matter (PM_2.5_) pollution and its action mechanism in the Sichuan–Chongqing urban agglomeration were considered for both socioeconomic and natural factors. The results showed that the “pollution halo” hypothesis of FDI in the Sichuan–Chongqing urban agglomeration has been supported. There are significant positive spatial spillover effects of PM_2.5_ pollution in this urban agglomeration, and the introduction of FDI is conducive to alleviating PM_2.5_ pollution in the urban agglomeration. Similar to the “inverted U” curve proposed by the environmental Kuznets curve (EKC) hypothesis, there was a significant “inverted U” curve relationship between PM_2.5_ pollution and economic growth in the Sichuan–Chongqing urban agglomeration. However, there was a significant “U”-type curve relationship between the urbanization degree and the PM_2.5_ concentration, which indicates that the current urbanization mode may aggravate the pollution degree of PM_2.5_ in the urban agglomeration in the long term. Furthermore, the two natural factors of annual average temperature and annual precipitation play an important role in PM_2.5_ pollution and spatial spillover effect in the Sichuan–Chongqing urban agglomeration. Economic development and rationalization of the industrial structure are the main ways by which FDI affects PM_2.5_ pollution in the urban agglomeration. The research conclusions of this study can be of great practical significance to optimize the regional industrial layout, control PM_2.5_ pollution, and establish a sustainable development policy system in the Sichuan–Chongqing urban agglomeration.

## 1. Introduction

The Sichuan–Chongqing urban agglomeration, located in the western region of China, was the key area of the “third-line construction” in 1964, a period of early large-scale industrial migration in the economic history of New China. It was during this special period that China’s central government constructed more than three hundred large-scale industrial projects in Sichuan and Chongqing—including the military, metallurgy, machinery, chemical industry, petroleum, and steel industries—which provided a unique foundation for the industrial development of China’s urban agglomeration [1,2]. After years of development, the Sichuan–Chongqing urban agglomeration has developed into the most densely populated urban agglomeration in western China. In recent years, China’s central government has issued a series of policies to support the development of the city cluster. Under the influence of these preferential regional development policies and a solid heavy industry foundation, the Sichuan–Chongqing urban agglomeration has attracted a large number of foreign investments in recent years. Among this urban agglomeration, Chengdu has been ranked first as “China’s most attractive city for investment” for nine consecutive years. However, with the rapid social and economic development of the Sichuan–Chongqing urban agglomeration, the problem of air pollution prevention and control has become serious. Particulate matter (PM_2.5_) pollution is the most significant form of air pollution in this region [3,4,5]. Meteorological satellite monitoring data show that the annual average of PM_2.5_ pollution in the Sichuan–Chongqing urban agglomeration in 2021 was 33.4 μg/m^3^, 1.11 times China’s average level (30 μg/m^3^) and 1.35 times the World Health Organization’s target limit (25 μg/m^3^). Studies have shown that PM_2.5_ pollution is a major threat to human health, causing respiratory diseases and even cancer [6,7]. Furthermore, the estimated number of deaths attributable to PM_2.5_ pollution in China was 971,000 in 2017 [8].

From the perspective of economic development, pollution intensification is closely related to many negative economic factors, such as extensive development, the excessively high proportion of coal consumption, the out-of-date industrial structures, and the low environmental governance efficiency [9]. According to the literature, the economic development path of a region often has path dependence. In particular, the cumulative effects of investment on advantageous industries cause the structural transformation of urban development to face resistance from the cumulative effects. Foreign direct investment (FDI) is an important aspect of investment, and scholars worldwide have expressed concerns about its environmental effects [10]. In recent years, the literature on this issue has become increasingly rich. Two opposing hypotheses on the issue are “pollution halo” and “pollution haven”. The “pollution halo” hypothesis involves a positive evaluation of the environmental effect of FDI based on the technical spillover effect brought about by FDI. Some scholars believe that multinationals investing in developing countries can help them improve environmental production by spreading cleaner and green production technologies to host countries [11,12]. In contrast, the “pollution halo” hypothesis focuses more on the negative impact of FDI on the transfer of high pollution [13,14] and energy consumption industries [15,16] to developing countries. A literature search indicates that the following question needs to be further explored: whether the rapid growth of FDI in the Sichuan–Chongqing urban agglomeration promotes local PM_2.5_ pollution or whether local PM_2.5_ pollution is alleviated through FDI’s technical spillover effect. Existing studies have not yet provided clear empirical evidence and mechanistic explanations for this question.

A large number of studies show that the change process of air pollution in urban agglomerations has obvious regional, compound, and cumulative characteristics and that many complex factors lead to pollution [17]. From a meteorological point of view, in addition to pollution emission—which is considered the root cause of heavy air pollution—natural factors such as meteorological conditions and complex terrain have an important impact on the transmission, diffusion, and accumulation of pollutants [18]. At the same time, although urbanization has produced an agglomeration effect and promoted economic development, the rapidly expanding urban scale has also brought about “urban diseases” such as traffic congestion and environmental pollution [19]. Many factors such as economic scale, industrial structure, and production technology affect the impact of FDI on the environment [20]. Historically, the Sichuan–Chongqing urban agglomeration, which can develop rapidly only on a resource-based industrial foundation, has witnessed large energy consumption. It is located in the Sichuan Basin, which has a special terrain susceptible to adverse meteorological factors, leading to severe pollution [21]. Therefore, after controlling for economic and natural factors, it is of great practical significance to explore the influence mechanism of FDI on PM_2.5_ pollution in urban agglomerations, comprehend the evolution of PM_2.5_ pollution with FDI development, and then optimize the regional industrial layout, control PM_2.5_ pollution, and establish a sustainable development policy system for the Sichuan–Chongqing urban agglomeration.

This paper used panel data of nineteen key cities in the Sichuan–Chongqing urban agglomeration from 2003 to 2016 as the research sample; explored the influence of FDI on PM_2.5_ pollution in the Sichuan–Chongqing urban agglomeration; and clarified the influence mechanism of FDI on PM_2.5_ pollution in the Sichuan–Chongqing urban agglomeration. There are four major innovations in this paper: (a) urban agglomeration was the research object—specifically the Sichuan–Chongqing urban agglomeration—and data of prefecture-level cities were used to capture the spatial effect of air pollution more precisely; (b) this study included natural factors as control variables in the model so that its empirical results are more reliable than those of traditional studies that focus only on economic variables; (c) regarding industrial structure, we selected two indicators, advanced industrial structure and industrial structure rationalization, to depict the industrial structure of the Sichuan–Chongqing urban agglomeration, which was helpful in further clarifying the action mechanism of the industrial structure; and (d) three indicators—the light composite index constructed by global night light data combined with the park green area and public transportation situation—were used to measure the urbanization level of the Sichuan–Chongqing urban agglomeration, thereby avoiding the possible statistical error caused by an urbanization rate that only considers the representation of the urban population proportion.

The rest of this paper is organized as follows: Section 2 presents the study area, theoretical models, and data sources; Section 3 presents the empirical results; Section 4 presents the discussion; and Section 5 presents the conclusions and policy implications.

## 2. Materials and Methods

### 2.1. Study Area

**Figure 1:**
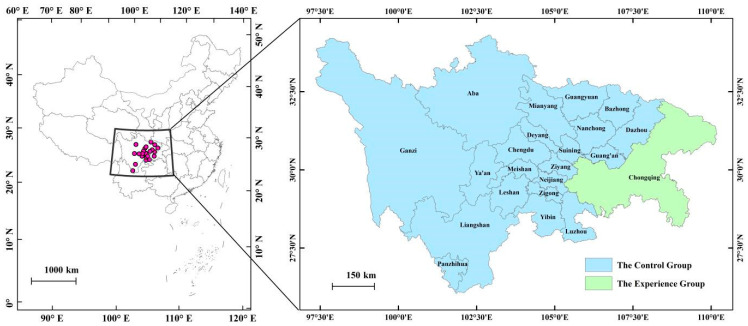
The Sichuan–Chongqing urban agglomeration in China.

The Sichuan–Chongqing urban agglomeration is located in the upper reaches of the Yangtze River in southwest China; moreover, the Sichuan province and Chongqing municipality can be divided into two natural geographical units: the eastern basin area and the western plateau area (Figure 1). In terms of natural conditions, the economy, and society, the Sichuan–Chongqing urban agglomeration is significantly different from the urban agglomerations in the eastern region of the Beijing–Tianjin–Hebei region, the Yangtze River Delta, and the Pearl River Delta. The differences are mainly reflected in four aspects: (a) the Sichuan–Chongqing urban agglomeration is located in the Sichuan Basin, and its marginal relative height difference is 500~2500 m, whereas the urban agglomeration in the eastern region is mainly located in the plain area; (b) although the Sichuan–Chongqing urban agglomeration belongs in the subtropical monsoon humid climate zone, its climate is similar to the temperate marine climate—rainy and foggy all year round—whereas the urban agglomeration in the eastern region has a typical temperate monsoon climate; (c) the per capita GDP of the Sichuan–Chongqing urban agglomeration is relatively low based on 2021 data, which indicate that the per capita GDP of the Sichuan–Chongqing urban agglomeration was 75,600 yuan in 2021—far lower than that of the Beijing–Tianjin–Hebei urban agglomeration (117,300 yuan), the Yangtze River Delta urban agglomeration (123,500 yuan), and the Pearl River Delta urban agglomeration (153,000 yuan); and (d) the Sichuan–Chongqing urban agglomeration is still in the medium-term stage of urbanization development based on 2021 data, which indicate that the urbanization rate of the Sichuan–Chongqing urban agglomeration was 64.06% in 2021—lagging behind the Beijing–Tianjin–Hebei urban agglomeration (65.8%), the Yangtze River Delta (73.83%), and the Pearl River Delta (80%). Additionally, many studies have proved that because of the abovementioned historical reasons, the current industrial structure rationalization degree and optimization speed of the Sichuan–Chongqing urban agglomeration lag behind those of the urban agglomeration in the eastern region. At the same time, although the industrial structure of the Sichuan–Chongqing urban agglomeration is gradually becoming more advanced, the advanced stability is still insufficient compared with that of the urban agglomerations in eastern China [22,23].

### 2.2. Model

As an effective method to quantitatively analyze environmental load, the stochastic impacts by regression on population, affluence, and technology (STIRPAT) model has been widely used in environmental protection research [24,25,26]. The STIRPAT model originated from the IPAT equation [27]:

(1) I=P·A·T

where *I* is environmental load, *P* is population size, *A* is affluence, and *T* is technical level.

To overcome the deficiency of the IPAT equation, York et al. constructed a STIRPAT model based on the IPAT equation [25]:

(2) $$I=aP^{b}A^{c}T^{d}\varepsilon$$

where *a* is a constant; *b*, *c*, and *d* are the index terms of *P*, *A*, and *T*, respectively; and ε is an error term. Equation (2) is transferred from the logarithmic process to the linear expansion model [28] as follows:

(3) lnI=lna+blnP+clnA+dlnT+lnε

In the process of studying environmental load by using the STIRPAT model, other factors can be introduced to affect environmental load according to the actual situation of the study area [29,30]. To explore the effect of FDI on PM_2.5_ pollution in the Sichuan and Chongqing region, we constructed an extended STIRPAT model logarithmic based on the study of Zhu and Jiang [31,32]:

(4) $$lnPM_{2.5}=lna+b\ln P_{it}+c\ln A_{it}+d\ln T_{it}+e\ln FDI_{it}+h\ln X_{it}+\mu_{i}+\nu_{t}+\varepsilon_{it}$$

where *PM*_2.5_ is urban PM_2.5_ pollution; *P* represents population density; *A* represents economic development level, measured by GDP; *T* represents technology and power energy efficiency [33,34]; *FDI* is measured by foreign investment amount; *X* is the control variable group; *a* is the constant, and *b*, *c*, *d*, *e*, *f*, *g,* and *h* are the coefficients; *i* is the city; *t* is the year; *u_i_* represents the fixed effect of city *i* that controls for features that do not change over time; *v_t_* is the annual fixed effect, used to control the time-varying omitted variables and random shocks common to all cities; and $\varepsilon_{it}$ is the error term.

The control variable group comprised two major categories—socioeconomic factors and natural factors—and we used ten indicators to interpret them. The socioeconomic factors included two parts: industrial structure and urbanization degree. The measurement method of industrial structure upgrading index (US) is to divide the output value of the tertiary industry by the output value of the secondary industry [35]. This index can reflect whether the industrial structure is developing toward a “service-oriented” direction. When the measurement results of industrial structure upgrading rise, this shows that the current economic structure is developing toward the service-oriented direction. Industrial structure rationalization (RS) is a measure of the coupling between an economy’s output and its factor input. This index jointly reflects the degree of rationalization of the industrial structure of the urban agglomeration from the two aspects of the coordination degree between industries and the effective utilization degree of resources. The measure of industrial structure rationalization index was based on the research method of Jia et al. [36]. The specific empirical model is shown in formula (5). The degree of urbanization includes the data of stable light, the park green area and the total amount of public transportation [37,38]. Natural factors include the average annual temperature, average annual precipitation, average annual relative humidity, average annual sunshine hours, and average annual wind speed [39,40].

The calculation formula of the industrial structure rationalization index is as follows:

(5) $$RS=\sum_{i=1}^{n}\frac{O_{i}}{O}\times \frac{O_{i}/L_{i}}{O/L}=\sum_{i=1}^{n}\frac{O_{i}}{O}\times \frac{O_{i}/O}{L_{i}/L}$$

where *O* is the output value; *L* is the labor force; *i* represents the *i*th industry; and *n* is the number of industrial sectors.

Considering the spatial spillover effect of the dependent variable PM_2.5_ (Appendix A contains a spatial autocorrelation test) and the lag effect on time, a dynamic spatial measurement model was constructed on the basis of Equation (4) [41]. The specific model is as follows:

(6) $$\ln PM_{2.5it}=\tau \ln PM_{2.5it-1}+\rho \overset{N}{\underset{j=1}{\sum }}w_{ij}\ln PM_{2.5jt}+b\ln P_{it}+c\ln A_{it}+d\ln T_{it}+e\ln FDI_{it}+h\ln X_{it}+\mu_{i}+\nu_{t}+\varepsilon_{it}$$

where *w* is the spatial weight matrix, and common spatial weight matrices may be a 0–1 matrix, an inverse distance matrix, an economic geography matrix, and so forth. To better investigate the spatial correlation characteristics of PM_2.5_ and consider the endogenous problem of the economic distance matrix, we used the inverse distance matrix as the spatial weight matrix. Here, *ρ* is the spatial factor of the dependent variable, and *τ* is the dynamic factor of the dependent variable [42].

### 2.3. Data Sources

Based on their availability and completeness, we used data from nineteen major cities in the 2003–2016 period. The PM_2.5_ concentration data were obtained from the Social and Economic Data and Application Center at Columbia University [43,44]. Stable light data were obtained from the US National Oceanic and Atmospheric Administration [45,46]. The data for the remaining variables in this paper were obtained from China Urban Statistical Yearbook (2003–2016) [47]. We divided the secondary and tertiary industries according to the standard of Industrial Classification of China’s National Economy (GB/T4754-2011). Table 1 shows the descriptive statistics of variables. GDP was based on 2002 data adjusted using the GDP deflator, which is the actual GDP excluding price changes.

Because the data used in this paper were short panel data, we referred to Harris and Tzavalis’s method to test unit roots [48]. Table 2 shows the test results. In the case of horizontal values, three variables were nonstable. After the first order difference of all variables, all the data sequences were stable.

Because many independent variables were involved in this study, we referred to Kao’s method [49]. Table 3 shows the test results, which significantly rejected the null hypothesis, indicating a stable, long-term equilibrium relationship between the independent variable and the control and dependent variables. Raw data were available for regression.

## 3. Results

### 3.1. Regression Results for the SDM

Based on the model setting, we used the spatial autoregressive model (SAR) and the spatial Durbin model (SDM) as the alternative models and analyzed them using Stata/SE16.0. The SDM model was first used to analyze the collected data with the Wald test and likelihood ratio test. The results revealed significant spatial spillover effects on the explanatory variables, thereby significantly rejecting the null hypothesis and indicating that the SDM model cannot be reduced to the SAR model. Furthermore, the results of the Hausman test significantly rejected the null hypothesis; therefore, we used the fixed-effect SDM model as the main tool for the empirical analysis.

In Table 4, columns (1)–(3) present the results of static SDM, and columns (4)–(6) present the results of dynamic SDM. We found that the time-lag factor was not significant after the addition of the natural factors, indicating that the PM_2.5_ concentration in the previous period was not significant in the current period. Through the Akaike information criterion (AIC) and the Bayesian information criterion (BIC) for model selection [50,51], we found the static model to be a better choice. Although the AIC and BIC indices of model (2) were smaller without adding control variables such as natural factors, the AIC and BIC values of model (3) were modest. Therefore, the subsequent analysis used model (3) as the benchmark model.

According to the regression results in Table 4, the estimates of all models indicated a negative effect of FDI on PM_2.5_, which in turn indicated that the introduction of FDI has been beneficial for alleviating PM_2.5_ pollution in the Sichuan–Chongqing urban agglomeration. It may have reduced the level of local pollution by improving local productivity or environmental technology or by spreading environmental awareness and sharing green management experience with local businesses. This finding is similar to that of Eskeland et al. [12]. Therefore, the “pollution halo” effect of FDI exists in the Sichuan–Chongqing urban agglomeration.

The primary coefficient of the economic development index was positive, the quadratic term coefficient was negative, and both were significant at 1%. This result indicated a significant “inverted U”-shaped curve relationship between PM_2.5_ pollution and economic growth. That is, the degree of PM_2.5_ pollution rises first with the economic growth level and then declines. The technical level had a significant negative impact on PM_2.5_ pollution (the significance level was 1%), indicating that the improvement in production technology can save resource consumption and reduce PM_2.5_ pollution. The impact of the industrial structure upgrading index on PM_2.5_ was not significant, showing that simply changing the industrial structure to a service one will not affect the environmental pollution in the Sichuan and Chongqing region. The rationalization index of the industrial structure had a significant positive impact on PM_2.5_ in the Sichuan–Chongqing urban agglomeration. This showed that the higher the rationalization index of an urban industrial structure, the more unreasonable the industrial structure is. This distorted allocation of essential factors may lead to the ineffective use of resources and aggravate the waste of urban resources and PM_2.5_ pollution caused by economic production. Different from the economic development index, a “U”-type curve relationship was found between the index of urbanization level and PM_2.5_ pollution. This is because in the early days of urbanization, there was a lot of demand for high-energy consumption products such as cement and steel, which led to massive energy consumption and environmental pollution [19]. In the middle and late stages of urbanization, with the promotion of green production and transportation such as landscaping and public transportation, urban PM_2.5_ pollution gradually develops in a positive direction. Among the natural factor control variables, the annual mean air temperature and annual precipitation had negative effects on PM_2.5_ pollution and were significant at a 12% significance level. This may be because, on the one hand, increased temperature helps inhibit aerosol formation and thus reduce PM_2.5_ pollution; on the other hand, increased precipitation helps reduce floating particles in the air, thereby reducing the degree of PM_2.5_ pollution. Population density and other natural factors were not significant.

### 3.2. Decomposition of Direct and Indirect Effects

When there is a spatial spillover effect, the change in a certain influencing factor not only causes PM_2.5_ pollution in the region but also affects PM_2.5_ pollution in the neighboring area and leads to a series of adjustment changes through circular feedback [9]. Therefore, referring to LeSage and Pace’s study, one step decomposed the effect of each factor on PM_2.5_ contamination into direct and indirect effects [52]. Because this paper used a dynamic spatial panel data model, we discussed empirical results of considering the long-term effects of time-lag effects.

Table 5 presents the impact effect decomposition results calculated based on the results presented in column (3) of Table 4. Both the direct and indirect effects of FDI were significantly negative, indicating that FDI agglomeration in the Sichuan–Chongqing urban agglomeration improves PM_2.5_ pollution in the surrounding areas. One possible explanation for is that, on the one hand, the employment opportunities brought by FDI cause a population migration and flow from the surrounding underdeveloped areas to developed areas, weakening the environmental pollution scale effect of population agglomeration in both areas. On the other hand, local governments have an imitation effect on “environmentally friendly” FDI under the pressure of performance assessments.

Both the direct and indirect effects at the technical level were significantly negative. This shows that the improvement in research and development strength in this region may help improve PM_2.5_ pollution in the surrounding areas; in other words, the improvement in production technology in this region may drive the improvement in energy-saving efforts in the surrounding areas through the spillover effect. This is because the industrial types within the urban agglomeration are often similar, which provides a realistic basis for the spillover effect of technology. It shows that in the early stage of economic development, the difficulty of PM_2.5_ pollution control in the Sichuan and Chongqing urban agglomeration and its surrounding areas may intensify. However, as the level of economic development continues to rise, PM_2.5_ pollution in this region and surrounding areas may decrease. The measurement results of the three indicators of the urbanization level showed that there is a significant “U”-type curve relationship between the urbanization degree and PM_2.5_ concentration, which indicates that the current urbanization mode will aggravate the degree of PM_2.5_ pollution in the Sichuan–Chongqing urban agglomeration in the long term. Moreover, because of the existence of the population “absorption effect,” the increase in local population will lead to the increase in local PM_2.5_ concentration and improve PM_2.5_ pollution in neighboring cities. Finally, the increase in green areas such as local parks may significantly reduce local PM_2.5_ concentration. Additionally, the improvement in energy efficiency will show a “demonstration effect” in the long term, which is conducive to PM_2.5_ pollution control in the surrounding cities, but the higher traffic intensity will significantly increase local PM_2.5_ pollution.

Among the natural factor control variables, the direct and indirect effects of annual mean temperature and annual precipitation were both negative and significant. These results prove that the climate characteristics of the Sichuan–Chongqing urban agglomeration—without extreme heat, severe cold, and perennial rain and fog—play an important role in the spread of PM_2.5_ pollution in the surrounding areas. However, the direct and indirect effects of the industrial structure upgrading index, population density, and other natural factors were not significant.

### 3.3. Robustness Test

#### 3.3.1. Replace the Measurement Method of the Industrial Structure

Because the impact of the industrial structure upgrading index on PM_2.5_ was not significant, which was not consistent with our theoretical expectations, it was necessary to conduct a robustness test to further determine whether the impact of the industrial structure upgrading index on PM_2.5_ is robust. We used the GDP ratio of the tertiary industry as the proxy variable. Column (2) in Table 6 presents the estimated result after the variable was replaced. The results showed that the degree of industrial structure upgrading was still not significant for PM_2.5_ pollution in Chinese cities, which is the same result presented in column (3) in Table 4. Thus, the estimated results of the industrial structure upgrading index were robust.

#### 3.3.2. Consider the Effect of Capital Accumulation

In economic growth research, it is important to consider the effect of capital accumulation and learning by doing [53]. As a form of capital, FDI also has an accumulation effect. Therefore, we considered the FDI’s first-order lag variable L.lnFDI in the control variables. Column (1) in Table 6 presents the estimated result that considers the accumulation effect of FDI. Compared with the results of column (3) in Table 4, the estimation results of the other variables were less different; thus, the previous results are still robust.

#### 3.3.3. Replace the Spatial Weights Matrix

Since the results of spatial measurement are sensitive to the type of spatial weights, we further used the inverse economic distance matrix to test robustness. The inverse economic distance matrix was calculated using the annual average GDP of each city from 2003 to 2016. The results in column (3) in Table 6 show that after adopting the inverse economic distance matrix, except for few control variables, the coefficients of core explanatory variables were still robust.

### 3.4. Analysis of the Mechanism of FDI Effect on PM_2.5_ Contamination

The results in column (3) of Table 4 show that the “pollution halo” of FDI in the Sichuan–Chongqing urban agglomeration exists. Further, the impact of FDI in the Sichuan–Chongqing urban agglomeration on local PM_2.5_ pollution may be realized through economic development and rationalization of the industrial structure. To further clarify the transmission pathway of the FDI effect on PM_2.5_ pollution in the Sichuan–Chongqing urban agglomeration, we referred to Baron and Kenny’s method to identify the above conduction pathways using the mediation effect model [54].

According to Table 7, the estimated coefficient of the FDI was significant at the 1% level when the economic development index was considered as the mediating variable. This result showed that the economic development of Sichuan–Chongqing urban agglomeration has a partial intermediary effect on the influence mechanism of FDI on PM_2.5_. Similarly, when the industrial structure rationalization indicators were considered as intermediary variables, the estimated coefficient of FDI was significant at the 10% level. This showed that the rationalization degree of the industrial structure in the Sichuan–Chongqing urban agglomeration has a partial intermediary effect on the influence mechanism of FDI on PM_2.5_. None of the estimated coefficients in Table 7 except the above two indicators were significant. Therefore, the following conclusion could be drawn: the two indicators of economic development and the rationalization of the industrial structure all play a partial intermediary role in the influence mechanism of FDI on PM_2.5_. The results also showed that the distorted factor allocation can not only restrain the efficiency of economic and social development but also aggravate resource waste and PM_2.5_ pollution of the Sichuan and Chongqing urban agglomeration. Thus, the economic development and rationalization of the industrial structure are the main ways in which FDI affects PM_2.5_ pollution in the Sichuan–Chongqing urban agglomeration.

## 4. Discussion

The Sichuan–Chongqing urban agglomeration is known as an important growth pole for high-quality economic development in western China. Because of the influence of the heavy industry transfer policy in the early days of the founding of the People’s Republic of China, the proportion of resource-based industries in the Sichuan–Chongqing urban agglomeration is significantly higher than that in urban agglomerations in eastern China. In recent years, under the policy guidance of central and local governments, the Sichuan–Chongqing urban agglomeration has attracted a large amount of foreign investment. However, this has led to a considerable PM_2.5_ pollution problem [3,4,5]. In the context of high-quality economic and ecological development, it is necessary to carry out research on the influence mechanism of FDI on PM_2.5_ pollution in the Sichuan–Chongqing urban agglomeration while considering both economic and natural factors. Based on the empirical results of this study, we found that the “pollution halo” hypothesis of FDI is supported in the Sichuan–Chongqing urban agglomeration. Moreover, the agglomeration of FDI in the Sichuan–Chongqing urban agglomeration can improve PM_2.5_ pollution in the surrounding areas. These results support the notion that FDI has important technical spillover effects, as Eskeland et al. suggested [11,12]. That is, “pollution heaven” and “pollution halo” are not unchanged; in the transition period of economic development, the FDI innovation effect may gradually be concentrated in the ecological innovation spillover effect. FDI, through the natural environment directly and through ecological technology innovation overflow indirectly, may reduce environmental pollution, indicating that the environmental effect of FDI meets the “pollution halo” hypothesis [55].

We also showed the same “inverted U” curve as the classical environmental Kuznets curve, indicating that there is a significant “inverted U” curve relationship between PM_2.5_ pollution and economic growth in the Sichuan–Chongqing urban agglomeration. Meanwhile, there was an opposite significant “U” curve relationship between the degree of urbanization and PM_2.5_ concentration. This finding demonstrates the long-term relationship between socioeconomic growth and pollution [56]. With the comprehensive development of the social economy, people tend to make higher demands regarding environmental quality, influence the government’s environmental regulation, and influence the production and pollution behavior of enterprises through their consumption preference and the pressure of public opinion [19]. Further, the process of urbanization produces positive externalities, thus reducing environmental damage through economies of scale, the agglomeration effect, the resource reallocation effect, and so on [57,58,59]. Compared with social and economic factors, natural factors play a more stable role in the influence of FDI on PM_2.5_ pollution in the Sichuan–Chongqing urban agglomeration.

## 5. Conclusions

In this paper, panel data from nineteen key cities in the Sichuan–Chongqing urban agglomeration from 2003 to 2016 were used as the study sample. Using the STIRPAT model, the effect of FDI on PM_2.5_ pollution and its action mechanism in the Sichuan–Chongqing urban agglomeration were considered for both socioeconomic and natural factors. The results showed that the “pollution halo” hypothesis of FDI in the Sichuan–Chongqing urban agglomeration is supported. There are significant positive spatial spillover effects of PM_2.5_ pollution in the Sichuan–Chongqing urban agglomeration, and the introduction of FDI is conducive to alleviating PM_2.5_ pollution in the urban agglomeration. Similar to the “inverted U” curve proposed by the classical EKC hypothesis, there was a significant “inverted U” curve relationship between PM_2.5_ pollution and economic growth in the Sichuan–Chongqing urban agglomeration. However, there was a significant “U”-type curve relationship between the urbanization degree and the PM_2.5_ concentration, which indicates that the current urbanization mode may aggravate the pollution degree of PM_2.5_ in the Sichuan–Chongqing urban agglomeration in the long term. The two natural factors of annual average temperature and annual precipitation play an important role in PM_2.5_ pollution and spatial spillover effect in the Sichuan–Chongqing urban agglomeration. Economic development and rationalization of the industrial structure are the main ways for FDI to affect PM_2.5_ pollution in the Sichuan–Chongqing urban agglomeration. Based on the conclusions of this study, we have three recommendations for local governments.

First, local governments should establish a reasonable management mechanism for foreign investment. While promoting the reform of foreign investment, the government should formulate and improve domestic foreign investment laws and regulations and relevant normative documents, attract more high-quality and clean foreign investment to the service industry, and promote the green upgrading and sustainable development of China’s manufacturing industry. Second, local governments should guide the rational development of industries. They should reasonably plan the urban industrial layout and jointly promote the intensive development of the Sichuan–Chongqing urban agglomeration by accelerating the adjustment of the urban industrial structure, promoting clean technology research and development, promoting the development of clean energy and improvement in energy efficiency, and rationally planning the urban transportation system. They should strengthen the improvement and management of industries with high emissions and high pollution and eliminate the illegal discharge of pollutants. They should change the economic development mode and force the green upgrading of the industrial structure and energy structure through market environmental regulation. They should actively seek alternative energy solutions, adjust the energy structure, and improve the energy quality so as to promote the upgrading of the industrial structure. Third, they should establish a joint PM_2.5_ pollution control and prevention mechanism. The spatial spillover effect and regional agglomeration characteristics of PM_2.5_ pollution indicate that the effective prevention and control of PM_2.5_ must be based on regional joint prevention and control. Local governments should implement PM_2.5_ pollution control as a global, long-term development strategy and implement the atmospheric environment function area as a unit; use organization and system resources to break the boundaries of administrative areas; establish a PM_2.5_ regional zone-spreading mechanism and joint planning and implementation of PM_2.5_ pollution control schemes; and implement regional PM_2.5_ pollution information sharing and joint early warnings. The government, enterprises, and residents should strive for collaborative governance of PM_2.5_ pollution.

One limitation of this study is that PM_2.5_ contamination is the result of the interaction between human activities and natural factors. The change in PM_2.5_ pollution is related to not only overall regional FDI, urbanization level, industrial structure characteristics, and other factors but also the industrial distribution of FDI, the industrial agglomeration form, and the lifestyle of residents. Our future research will combine the impact of the abovementioned data on PM_2.5_ with subdivided industry data, monthly meteorological data, and animal and plant data to build a more reasonable indicator system.

## Figures and Tables

**Table 1:** Variables in the empirical model.

Variable		Meaning	Obs	Mean	Std. Dev.	Min	Max
Explained variable	*PM*_2.5_	PM_2.5_ (μg/m^3^)	266	33.472	9.673	9.967	59.133
Explanatory variables	*P*	Population density (million people/km^2^)	266	770.896	446.558	199.383	2677.716
	*A*	GDP (million yuan)	266	614,000	973,000	102,000	5,510,000
	*T*	GDP per unit of electricity (yuan/billion kwh)	266	1.878	1.459	0.261	12.513
	*FDI*	Foreign investment amount (million yuan)	266	49,819.79	173,000	0	955,000
Control variables	*US*	Industrial structure upgrading index	266	0.688	0.285	0.272	1.955
	*RS*	Industrial structure rationalization index	266	4.117	8.373	1.008	85.086
	*Light*	Stable light	266	4.06	3.601	0.042	20.397
	*PGA*	Park green space area (hectares)	266	1426.474	3510.039	43.483	24,504.99
	*PTR*	Total bus and tram traffic volume (million people)	266	22,108.54	43,601.75	1	242,000
	*AT*	Average temperature (℃)	266	17.817	1.122	15.9	22.1
	*Pre*	Precipitation (mm)	266	1027.681	265.337	537.7	2092.3
	*ARH*	Average relative humidity (%)	266	75.163	6.519	48.58	88
	*SH*	Sunshine hours (h)	266	1177.743	424.019	598.4	2939.1
	*AWP*	Average wind speed (m/s)	266	1.507	0.151	1.1	1.8

**Table 2:** Unit root test.

Variables	Level Value			First Difference Value		
	Rho	*Z* Value	*p* Value	Rho	*Z* Value	*p* Value
*lnPM_2.5_*	0.0509	−15.9376	0.0000	−0.5116	−25.8310	0.0000
*l* *nA*	0.7909	−0.1930	0.4235	0.0491	−14.6669	0.0000
*l* *nP*	0.6568	−3.0468	0.0012	−0.1464	−18.5601	0.0000
*lnT*	0.6230	−3.7651	0.0001	−0.0986	−17.6089	0.0000
*lnFDI*	0.2779	−11.1087	0.0000	−0.3329	−22.2739	0.0000
*lnUS*	0.8574	1.2212	0.8890	0.1241	−13.1729	0.0000
*lnRS*	0.6615	−2.9469	0.0016	−0.1374	−18.3813	0.0000
*l* *nLight*	0.6418	−3.3659	0.0004	−0.7750	−31.0768	0.0000
*l* *nPGA*	0.7869	−0.2787	0.3902	0.0769	−14.1132	0.0000
*l* *nPTR*	0.3434	−9.7150	0.0000	−0.1806	−19.2407	0.0000
*l* *nAT*	0.0076	−16.8600	0.0000	−0.4752	−25.1060	0.0000
*l* *nPre*	−0.0438	−17.9543	0.0000	−0.4663	−24.9292	0.0000
*l* *nARH*	0.2710	−11.2550	0.0000	−0.3364	−22.3434	0.0000
*l* *nSH*	−0.0023	−17.0703	0.0000	−0.4719	−25.0414	0.0000
*l* *nAWP*	0.0258	−16.4716	0.0000	−0.4034	−23.6769	0.0000

**Table 3:** Cointegration test.

Test Method	Null Hypothesis	Statistics	Statistic Value	*p* Value
Kao test	*H*_0_: *ρ* = 1	DF	−9.3023	0.0000
		ADF	−4.5331	0.0000

**Table 4:** Regression results for the SDM.

	Static Model			Dynamic Model		
	(1)	(2)	(3)	(4)	(5)	(6)
*lnFDI*	−0.0130 ***	−0.0117 **	−0.0119 **	−0.0088 *	−0.0101 *	−0.0100 *
	(0.0047)	(0.0051)	(0.0050)	(0.0051)	(0.0056)	(0.0056)
*lnP*	0.0095	−0.0556	−0.0682	0.0524	−0.0114	−0.0273
	(0.0450)	(0.0478)	(0.0480)	(0.0484)	(0.0516)	(0.0522)
*lnA*	−0.1308 **	1.0106 **	1.0941 ***	−0.1663 ***	0.6329	0.7305
	(0.0530)	(0.3935)	(0.3979)	(0.0553)	(0.4364)	(0.4453)
*lnA* ^2^		−0.0394 ***	−0.0419 ***		−0.0271 *	−0.0299 *
		(0.0144)	(0.0146)		(0.0157)	(0.0161)
*lnT*	−0.0082	−0.0517 ***	−0.0538 ***	0.0026	−0.0345 *	−0.0326 *
	(0.0156)	(0.0170)	(0.0173)	(0.0167)	(0.0188)	(0.0195)
*lnUS*		0.0264	0.0352		−0.0015	0.0059
		(0.0441)	(0.0444)		(0.0476)	(0.0487)
*lnRS*		0.0272 *	0.0337 **		0.0190	0.0252
		(0.0151)	(0.0152)		(0.0157)	(0.0158)
*lnLight*		0.0836 ***	0.0756 ***		0.0746 ***	0.0699 ***
		(0.0224)	(0.0235)		(0.0232)	(0.0245)
*lnLight* ^2^		0.0188 **	0.0170 **		0.0172 **	0.0159 *
		(0.0081)	(0.0084)		(0.0083)	(0.0086)
*lnPGA*		−0.0188	−0.0218		−0.0264	−0.0270
		(0.0194)	(0.0193)		(0.0225)	(0.0224)
*lnPTR*		−0.0133	−0.0131		−0.0088	−0.0078
		(0.0092)	(0.0093)		(0.0146)	(0.0153)
*lnAT*			−0.5473 ^△^			−0.3718
			(0.3471)			(0.3589)
*lnPre*			−0.0503 ^△^			−0.0456
			(0.0316)			(0.0328)
*lnARH*			−0.0761			−0.0362
			(0.1510)			(0.1529)
*lnSH*			0.0222			−0.0032
			(0.0549)			(0.0553)
*lnAWP*			−0.0413			−0.0145
			(0.0632)			(0.0649)
τ				0.1607 ***	0.0716 *	0.0350
				(0.0361)	(0.0386)	(0.0437)
ρ	793.3027 ***	706.4897 ***	685.5686 ***	798.8251 ***	721.9586 ***	690.2247 ***
	(25.1684)	(39.2044)	(41.9395)	(26.2848)	(38.7671)	(44.1862)
sigma2_e	0.0054 ***	0.0045 ***	0.0044 ***	0.0053 ***	0.0047 ***	0.0046 ***
	(0.0005)	(0.0004)	(0.0004)	(0.0005)	(0.0004)	(0.0004)
Hausman P	0.0081					
AIC	−567.6976	−599.656	−592.3807	−543.2949	−556.8933	−549.1694
BIC	−531.8626	−513.652	−470.5419	−504.6916	−469.1586	−426.3408
Wald test P	0.1197	0.0032	0.0009	0.0251	0.0508	0.0264
Lratio test P	0.5577	0.0000	0.0000	0.5704	0.0060	0.0060
R-squared	0.3250	0.3484	0.3529	0.3378	0.3521	0.3589
Observations	266	266	266	247	247	247
Number of cities	19	19	19	19	19	19

Notes: Standard errors in parentheses; *** represents the significance at the 1% level; ** represents the significance at the 5% level; * represents the significance at the 10% level; ^△^ represents the significance at the 12% level; and superscript 2 represents the square of the variable.

**Table 5:** Decomposition of effects in the dynamic SDM model.

	LR_Direct	LR_Indirect	LR_Total
*lnFDI*	−0.0217 ***	−0.1280 *	−0.1496 **
	(0.0079)	(0.0669)	(0.0732)
*lnP*	−0.0906	−0.3081	−0.3987
	(0.0868)	(0.8190)	(0.8948)
*lnA*	2.6620 ***	20.4486 ***	23.1106 ***
	(0.7354)	(7.0671)	(7.7089)
*lnA* ^2^	−0.0977 ***	−0.7294 ***	−0.8270 ***
	(0.0276)	(0.2635)	(0.2878)
*lnT*	−0.1026 ***	−0.6304 ***	−0.7329 ***
	(0.0254)	(0.1957)	(0.2157)
*lnUS*	0.0618	0.3580	0.4198
	(0.0454)	(0.3321)	(0.3540)
*lnRS*	0.0658 ***	0.4360 **	0.5019 **
	(0.0230)	(0.1806)	(0.1991)
*lnLight*	0.1357 ***	0.7676 ***	0.9033 ***
	(0.0267)	(0.2040)	(0.2191)
*lnLight* ^2^	0.0179	0.0114	0.0294
	(0.0109)	(0.0818)	(0.0894)
*lnPGA*	−0.0430 **	−0.2698 **	−0.3128 **
	(0.0241)	(0.1790)	(0.1943)
*lnPRT*	−0.0453 **	−0.4093 **	−0.4545 **
	(0.0189)	(0.1767)	(0.1939)
*lnAT*	−0.2738	3.1828 **	2.9090 **
	(0.3652)	(1.2412)	(1.3274)
*lnPre*	−0.0586 *	−0.0815 *	−0.1401
	(0.0329)	(0.1568)	(0.1694)
*lnARH*	0.0566	1.5475	1.6041
	(0.1759)	(1.1132)	(1.2090)
*lnSH*	−0.0057	−0.3078	−0.3135
	(0.0558)	(0.2485)	(0.2711)
*lnAWP*	−0.0176	0.3028	0.2852
	(0.0905)	(0.5763)	(0.6465)

Notes: Standard errors in parentheses; *** represents the significance at the 1% level; ** represents the significance at the 5% level; * represents the significance at the 10% level; superscript 2 represents the square of the variable.

**Table 6:** Robustness test.

	(1)	(2)	(3)
*lnFDI*	−0.0115 **	−0.0128 **	−0.0141 *
	(0.0050)	(0.0050)	(0.0082)
*L.lnFDI*	−0.0068		
	(0.0045)		
*lnP*	−0.0638	−0.0623	−0.2507 ***
	(0.0478)	(0.0482)	(0.0782)
*lnA*	1.1311 ***	0.8353 **	0.3828
	(0.3961)	(0.3613)	(0.6862)
*lnA* ^2^	−0.0423 ***	−0.0328 **	−0.0222
	(0.0145)	(0.0134)	(0.0250)
*lnT*	−0.0540 ***	−0.0539 ***	−0.1002 ***
	(0.0172)	(0.0174)	(0.0281)
*lnUS*	0.0265	−0.0426	−0.2168 ***
	(0.0450)	(0.0816)	(0.0600)
*lnRS*	0.0368 **	0.0373 **	0.0771 ***
	(0.0152)	(0.0152)	(0.0232)
*lnLight*	0.0787 ***	0.0687 ***	0.1066 ***
	(0.0234)	(0.0236)	(0.0364)
*lnLight* ^2^	0.0186 **	0.0155 *	0.0609 ***
	(0.0084)	(0.0084)	(0.0129)
*lnPGA*	−0.0155	−0.0229	−0.1081 ***
	(0.0198)	(0.0193)	(0.0302)
*lnPTR*	−0.0125	−0.0140	−0.0296
	(0.0093)	(0.0093)	(0.0196)
*lnAT*	−0.4644	−0.4809	0.1371
	(0.3470)	(0.3496)	(0.3726)
*lnPre*	−0.0490	−0.0450	−0.0365
	(0.0314)	(0.0320)	(0.0429)
*lnARH*	−0.0807	−0.0542	−0.2367
	(0.1501)	(0.1523)	(0.2198)
*lnSH*	0.0304	0.0180	0.0866
	(0.0547)	(0.0549)	(0.0730)
*lnAWP*	−0.0328	−0.0474	0.0640
	(0.0631)	(0.0633)	(0.1019)
ρ	674.1420 ***	683.0380 ***	49.6225 **
	(43.3322)	(42.5661)	(21.8412)
sigma2_e	0.0043 ***	0.0044 ***	0.0118 ***
	(0.0004)	(0.0004)	(0.0010)
AIC	−593.1231	−590.7814	−355.7480
BIC	−464.1172	−468.9425	−233.9092
Wald test P	0.0008	0.0012	0.0001
Lratio test P	0.0000	0.0000	0.0003
R-squared	0.3554	0.3532	0.0586
Observations	266	266	266
Number of cities	19	19	19

Notes: Standard errors in parentheses; *** represents the significance at the 1% level; ** represents the significance at the 5% level; * represents the significance at the 10% level; superscript 2 represents the square of the variable.

**Table 7:** Empirical results of the mechanism of FDI effects on PM_2.5_ contamination.

	Lngdp	Lnpop	Lneff	Lnus	Lnrs	Lnlight
*lnFDI*	0.0224 ***	0.0121 *	0.0005	−0.0179 **	−0.0383 *	0.0163
*lnP*	−0.1244 **		0.0783	0.1361 **	−0.4465 **	−0.1661
	(0.0516)		(0.1715)	(0.0667)	(0.1931)	(0.1729)
	(0.0056)	(0.0064)	(0.0180)	(0.0070)	(0.0201)	(0.0180)
*lnA*		2.2017 ***	2.0917	−4.6812 ***	4.6806 ***	−5.4028 ***
		(0.4924)	(1.4167)	(0.4805)	(1.5822)	(1.3414)
*lnA* ^2^		−0.0876 ***	−0.0844	0.1648 ***	−0.1788 ***	0.1935 ***
		(0.0179)	(0.0519)	(0.0179)	(0.0578)	(0.0493)
*lnT*	−0.0152	0.0114		−0.0078	−0.0717	0.2384 ***
	(0.0195)	(0.0221)		(0.0241)	(0.0697)	(0.0610)
*lnUS*	−0.1299 ***	0.0795	−0.0014		−0.1060	−0.1456
	(0.0423)	(0.0568)	(0.1580)		(0.1795)	(0.1616)
*lnRS*	−0.0075	−0.0471 **	−0.0624	−0.0216		−0.1054 *
	(0.0166)	(0.0192)	(0.0538)	(0.0212)		(0.0548)
*lnLight*	−0.0339	0.0075	0.1576 *	−0.0455	−0.1579 *	
	(0.0252)	(0.0303)	(0.0835)	(0.0328)	(0.0951)	
*lnLight* ^2^	−0.0066	0.0191 *	−0.0365	−0.0112	−0.0111	
	(0.0092)	(0.0107)	(0.0299)	(0.0117)	(0.0339)	
*lnPGA*	−0.0001	0.0109	−0.1743 ***	−0.0305 **	−0.0363	−0.0382
	(0.0106)	(0.0119)	(0.0308)	(0.0130)	(0.0375)	(0.0332)
*lnPRT*	0.0698 ***	−0.0899 ***	−0.1603 **	0.0181	0.0471	−0.2350 ***
	(0.0211)	(0.0238)	(0.0677)	(0.0270)	(0.0782)	(0.0665)
*lnAT*	−0.3944	−1.0800 **	−2.2710 *	1.0195 **	−0.1035	−3.1486 **
	(0.3913)	(0.4418)	(1.2373)	(0.4838)	(1.4050)	(1.2519)
*lnPre*	0.0871 **	−0.0075	0.0489	0.0659	0.0711	0.1587
	(0.0357)	(0.0406)	(0.1132)	(0.0442)	(0.1282)	(0.1150)
*lnARH*	−0.0788	−0.0341	−0.8353	0.3523 *	−0.2703	−0.9075 *
	(0.1684)	(0.1946)	(0.5393)	(0.2102)	(0.6013)	(0.5367)
*lnSH*	0.0472	0.0962	0.5730 ***	−0.1160	0.1737	0.0566
	(0.0623)	(0.0705)	(0.1934)	(0.0768)	(0.2234)	(0.2009)
*lnAWP*	−0.0049	0.0954	0.1959	−0.0586	0.0699	−0.5072 **
	(0.0714)	(0.0806)	(0.2260)	(0.0884)	(0.2570)	(0.2266)
ρ	263.2409 **	−166.7196	−209.0299 *	447.1121 ***	−147.9176	263.1785 **
	(102.5532)	(145.2695)	(125.1422)	(81.2307)	(128.3881)	(110.9647)
sigma2_e	0.0058 ***	0.0073 ***	0.0564 ***	0.0087 ***	0.0727 ***	0.0591 ***
	(0.0005)	(0.0006)	(0.0049)	(0.0008)	(0.0063)	(0.0052)
R-squared	0.0148	0.3973	0.1378	0.0822	0.0603	0.1259
Observations	266	266	266	266	266	266
Number of cities	19	19	19	19	19	19

Notes: ***, **, * indicate the significance levels of 1%, 5%, and 10%, respectively, and the values in the square brackets under the coefficients are the standard error.

**Figure A1:**
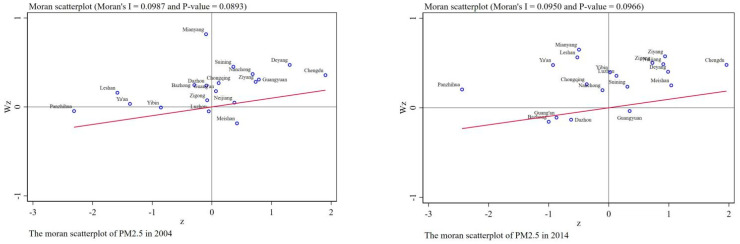
The scatter plot of PM_2.5_ pollution in Sichuan–Chongqing urban agglomeration in the geographic distance weight matrix.

## Appendix A

When the data involve geospatial features, the observed values cannot remain independent because the closer distance may cause relevance [60]. Related papers show that PM_2.5_ contamination can diffuse and transfer, causing interactions between neighboring cities. To conduct a comprehensive investigation of the spatial spillover effect of PM_2.5_ pollution in Sichuan–Chongqing urban clusters, Moran’s I index was used to test the spatial aggregation effect of PM_2.5_. The calculation formula is $I=[n\overset{n}{\underset{i=1}{\sum }}\overset{n}{\underset{j=1}{\sum }}w_{ij}\left(x_{i}-\bar{x}\right)\left(x_{j}-\bar{x}\right)]/[\overset{n}{\underset{i=1}{\sum }}\overset{n}{\underset{j=1}{\sum }}w_{ij}\overset{n}{\underset{i=1}{\sum }}\left(x_{i}-\bar{x}\right)]$, where *n* indicates the nineteen prefecture-level cities within the Sichuan–Chongqing urban agglomeration; *w_ij_* is the spatial weight; and *x* and the other variables are the PM_2.5_ concentration and its urban average value, respectively. The spatial correlation test results showed that Moran’s I index was greater than 0.6 and significant at the 1% level, indicating that the PM2.5 contamination distribution showed “high-high” and “low-low” spatial positive correlation characteristics. Therefore, it is necessary to consider the spatial econometric models that can reflect the spatial dependence of the sample data. As shown in Figure A1, a scatter plot of the PM_2.5_ concentration distribution in the Sichuan–Chongqing city clusters in two representative years is given, wherein the horizontal axis indicates the normalized PM_2.5_ concentration value and the vertical axis provides the spatial lag value of the PM_2.5_ concentration value.
